# The LACOG-0415 phase II trial: abiraterone acetate and ADT versus apalutamide versus abiraterone acetate and apalutamide in patients with advanced prostate cancer with non-castration testosterone levels

**DOI:** 10.1186/s12885-019-5709-y

**Published:** 2019-05-23

**Authors:** Gustavo Werutsky, Fernando Cotait Maluf, Eduardo Henrique Cronemberger, Vinicius Carrera Souza, Suelen Patricia dos Santos Martins, Fábio Peixoto, Oren Smaletz, Fábio Schutz, Daniel Herchenhorn, Telma Santos, Flavio Mavignier Carcano, David Queiroz Muniz, Paulo R. S. Nunes Filho, Facundo Zaffaroni, Carlos Barrios, André Fay

**Affiliations:** 1 Latin American Cooperative Oncology Group, Ipiranga Avenue 6681, 99A, Room, Porto Alegre, 806 Brazil; 2 Hospital São José, São Paulo, Brazil; 3 Centro Regional Integrado de Oncologia, Fortaleza, Brazil; 4 Clínica Assistência Multidisciplinar em Oncologia, Salvador, Brazil; 5 Centro de Estudos e Pesquisa de Hematologia e Oncologia, Santo André, Brazil; 6 Americas Centro de Oncologia Integrado, Rio de Janeiro, Brazil; 7 Hospital Israelita Albert Einstein, São Paulo, Brazil; 8 Beneficiência Portuguesa de São Paulo, São Paulo, Brazil; 9 Oncologia D’Or, Rio de Janeiro, Brazil; 10 Janssen Cilag Pharmaceutical, São Paulo, Brazil; 11 Hospital do Câncer de Barretos, Barretos, Brazil; 12 Instituto do Câncer do Estado de São Paulo, São Paulo, Brazil; 13 PUCRS School of Medicine, Porto Alegre, Brazil

**Keywords:** Castration-sensitive prostate cancer, Hormonal therapy, Androgen deprivation therapy, Abiraterone, Apalutamide, Goserelin

## Abstract

**Background:**

Testosterone suppression is the standard treatment for advanced prostate cancer, and it is associated with side-effects that impair patients’ quality of life, like sexual dysfunction, osteoporosis, weight gain, and increased cardiovascular risk. We hypothesized that abiraterone acetate with prednisone (AAP) and apalutamide, alone or in combination, can be an effective hormonal therapy also possibly decreasing castration-associated side effects.

**Methods:**

Phase II, open-label, randomized, efficacy trial of abiraterone acetate plus prednisone (AAP) and Androgen Deprivation Therapy (ADT) versus apalutamide versus the combination of AAP (without ADT) and apalutamide. Key eligibility criteria are confirmed prostate adenocarcinoma; biochemical relapse after definitive treatment (PSA ≥ 4 ng/ml and doubling time less than 10 months, or PSA ≥ 20 ng/ml); newly diagnosed locally advanced or metastatic prostate cancer; asymptomatic to moderately symptomatic regarding bone symptoms. Patients with other histology besides adenocarcinoma or previous use of hormonal therapy or chemotherapy were excluded.

**Discussion:**

There is an urgent need to study and validate regimens such as new hormonal agents that may add benefit to castration with an acceptable safety profile. We aim to evaluate if apalutamide in monotherapy or in combination with AAP is an effective and safety hormonal treatment that can spare patients of androgen deprivation therapy.

**Trial registration:**

This trial was registered in ClinicalTrials.gov on October 16, 2017, under Identifier: NCT02867020.

## Background

Patients with advanced prostate cancer are generally treated with surgical or chemical castration. Despite high response rates with this strategy, testosterone suppression is associated with libido loss, sexual dysfunction, hot flushes, osteoporosis, muscle weakness and weight gain [1]. Moreover, patients with metastatic prostate cancer are living longer as a result of several new life-prolonging treatments with good symptomatic control, most notably when androgen deprivation therapy is initiated early for rising prostate-specific antigen (PSA) after the front-line treatment for the primary tumor. Therefore, there is a need to investigate if other hormonal therapies that can robustly suppress androgen signaling may spare the side-effects typically associated with conventional castration [2–4].

Abiraterone acetate, which inhibits the key enzyme cytochrome P450 c17 (CYP17), prevents androgen production by testes, adrenal gland and the prostate tumor [5]. In Phase III clinical trials, AAP showed improved efficacy against placebo in patients with metastatic castration-resistant prostate cancer, pre and post-chemotherapy, along with an acceptable safety profile [6–8]. Moreover, AAP together with androgen deprivation therapy improved survival in patients with newly diagnosed, metastatic, castration-sensitive prostate cancer in the LATITUDE [9] and STAMPEDE trials [10].

Apalutamide is a second-generation antiandrogen that emerged from a structure/activity relationship–guided medicinal chemistry program to design more potent antiandrogens with no significant agonistic activity in the setting of AR overexpression [11]. A Phase II trial including 21 patients with castration-resistant prostate cancer who had failed prior abiraterone treatment has shown a response rate of 24% [11]. Additionally, co-targeting the androgen receptor and paracrine androgen biosynthesis in castration-resistant prostate cancer may be more effective than either alone. A Phase II study evaluated the activity of AAP and enzalutamide, another second-generation antiandrogen, at the conventional doses in 60 patients and reported a PSA decline ≥50% and ≥ 90% in 76 and 45% of patients, respectively, with an acceptable non-overlapping safety profile [12]. Additionally, another Phase II study [13] evaluated enzalutamide alone in hormone-naïve patients, without ADT, in 67 patients and shown a 92.5% PSA response rate (a decline of 80% or greater), regardless of metastases at baseline.

There is limited evidence for clinical application of these second-generation hormonal agents either alone or in combination in metastatic prostate cancer with non-castrate testosterone levels. In the phase III SPARTAN trial [14], apalutamide in combination with androgen deprivation therapy prolonged metastasis-free survival in men with nonmetastatic castration-resistant prostate cancer; noteworthy, apalutamide did not increase androgen suppression side effects as compared with placebo. As a result, apalutamide was approved in the United States in this setting.

## Methods/design

### Study design

**Fig. 1:**
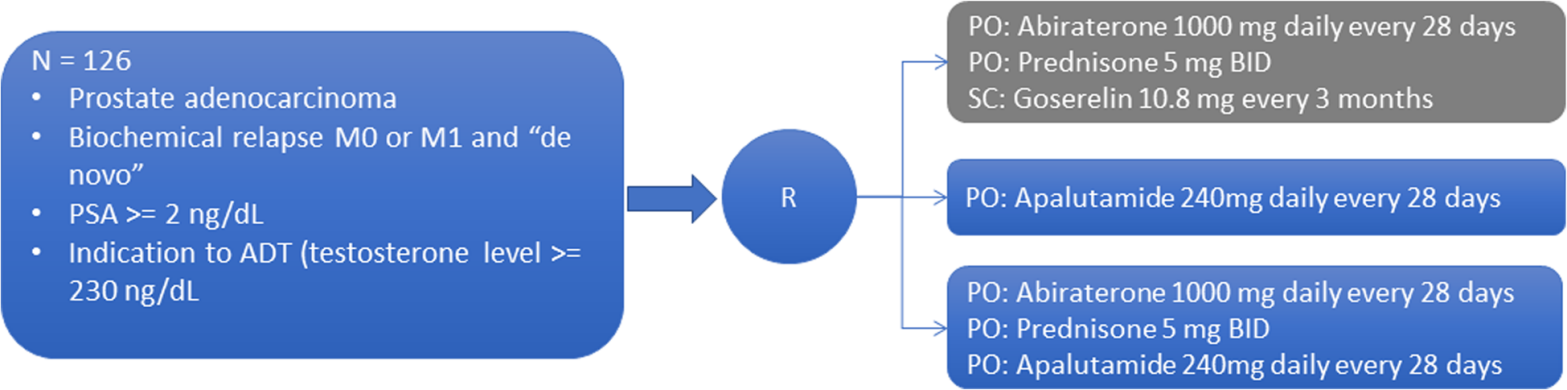
LACOG-0415 study design (schematic)

This is a phase II, open-label, randomized trial evaluating the efficacy of abiraterone acetate plus prednisone and Androgen Deprivation Therapy (ADT) versus apalutamide versus the combination of AAP (without ADT) and apalutamide, both at the standard doses, in patients with advanced or metastatic prostate cancer with non-castrate testosterone levels (Fig. 1). The total study period is 2 years including patient treatment and outcome data collection. Patients will be treated until objective or clinical disease progression or the occurrence of unacceptable toxicity. Patients are allowed to continue study treatment beyond the 25-week assessment (extension phase) at the discretion of the investigator. It will be conducted in 10 sites located in Brazil.

### Ethical considerations

The study protocol was reviewed and approved by the Institutional Review Board of all participating institutions (see details in Appendix 1). Written informed consent will be obtained from all participants by investigators before any study procedure. This trial was registered in ClinicalTrials.gov trial registry (NCT02867020).

### Inclusion criteria

Each potential subject must fulfill all of the following criteria to be enrolled in the study:

1. Histologically confirmed prostate adenocarcinoma;
2. Patients with indication to start treatment with ADT in one of the following settings:
  - Biochemical relapse after definitive front-line treatment (surgery and/or radiotherapy): PSA ≥ 4 ng/ml and doubling time less than 10 months, or PSA ≥ 20 ng/ml;
  - Newly diagnosed Prostate Cancer: locally advanced – Tany N+ M0 (not a candidate to definitive treatment with surgery or radiotherapy) or metastatic – Tany Nany M+ and PSA ≥ 2 ng/mL;
3. The patient is asymptomatic to moderately symptomatic regarding bone symptoms, i.e., no need for palliative radiation or radionuclide therapy;
4. Complete staging process (performed as per routine), meaning, thorax, abdomen and pelvis TC and bone scan, performed before consent and that do not exceed 10 weeks prior to the date of randomization
5. Non-castration level of testosterone ≥230 ng/dL (> 8 nmol/L);
6. ECOG performance status of 0 to 2;
7. Adequate hematologic, hepatic and renal function:
  - hemoglobin > 10 g/dL, neutrophils > 1.5 × 10^9^ / L, platelets> 100 × 10^9^ / L;
  - total bilirubin < 1.5x upper limit of normal (ULN); alanine (ALT) and aspartate (AST) aminotransferase < 2.5 x ULN;
  - serum creatinine < 1.5x ULN; potassium > 3.5 mM;
8. No previous cancer (except treated basal-cell skin cancer);
9. Written informed consent obtained prior to any study procedure;
10. Age 18 years and older;
11. Agrees to use a condom and another effective method of birth control if he is having sex with a woman of childbearing potential or agrees to use a condom if he is having sex with a woman who is pregnant

### Exclusion criteria

Any potential subject who meets any of the following criteria will be excluded from participating in the study.

1. Prostate adenocarcinoma with neuroendocrine differentiation or small cell histology;
2. Use of hormonal therapy or chemotherapy prior to randomization. The exception is courses of hormone therapy for localized disease, that must have been completed at least 12 months previously. It could have been given as adjuvant or neoadjuvant therapy.
3. Prior radiation therapy for a primary tumor within the 3 months before enrollment or for the treatment of metastases;
4. Known or suspected brain or skull metastases or leptomeningeal metastatic disease;
5. Any concurrent severe and/or uncontrolled medical conditions which could compromise participation in the study;
6. Administration of an investigational therapeutic or invasive surgical procedure within 28 days of Cycle 1 Day 1 or currently enrolled in an investigational study;
7. Active or symptomatic viral hepatitis or chronic liver disease; ascites or bleeding disorders secondary to hepatic dysfunction;
8. Current or prior treatment with anti-epileptic medications for the treatment of seizures;
9. Impaired cardiac function, including any of the following:
  - Uncontrolled hypertension (systolic blood pressure ≥ 160 mmHg or diastolic BP ≥95 mmHg);
  - Clinically significant heart disease as evidenced by myocardial infarction, or arterial thrombotic events or history of cardiac failure in the past 6 months, severe or unstable angina, or New York Heart Association (NYHA) Class II-IV heart disease;
  - Existing atrial fibrillation with or without pharmacotherapy. Other cardiac arrhythmia requiring pharmacotherapy;
10. History of seizure or condition that may predispose to seizure (including, but not limited to prior stroke, transient ischemic attack or loss of consciousness ≤1 year prior to randomization; brain arteriovenous malformation; or intracranial masses such as schwannomas and meningiomas that are causing edema or mass effect);
11. Specific underlying conditions for oral agents. For example impairment of gastrointestinal (GI) function or GI disease that may significantly alter the absorption of abiraterone acetate or apalutamide (e.g., ulcerative diseases, uncontrolled nausea, vomiting, diarrhea, malabsorption syndrome, or small bowel resection)
12. General excluded medications (e.g., relevant to cytochrome P450 interactions)
  - Use of prescription drugs within 14 days prior to dosing or over-the-counter (OTC) medication within 7 days prior to dosing;
  - Consumption of grapefruit product or St John’s wort within 7 days prior to dosing;
  - G-CSF, GM-CSF, erythropoietin, etc.;
  - Coumadin;
  - Drugs which may cause QT prolongation;
  - Known sensitivity to drugs or metabolites from similar classes;
  - Known or suspected contraindications or hypersensitivity to apalutamide, bicalutamide or GnRH agonists or any of the components of the formulations;
13. Any condition or situation which, in the opinion of the investigator, would put the subject at risk, may confound study results, or interfere with the subject’s participation in this study;
14. Surgical castration prior to study entry.

### Allocation and study treatment

Patients will be randomly assigned to three arms in a 1:1:1 ratio. The randomization will be balanced by using randomly permuted blocks. Randomization will take place across all study sites using a centralized Interactive Web Response System (IWRS). Subjects will be stratified by performance status (ECOG 0–1 vs 2) and metastatic disease (yes vs. no).

The study arms will be consisted of:

- Arm 1 (CONTROL): Abiraterone acetate + Prednisone + ADT (Goserelin).
  - Abiraterone acetate administered at a single 1000 mg daily oral dose (4 × 250 mg tablets)
  - Prednisone administered at a 5 mg twice daily oral dose
  - Goserelin administered as subcutaneous injections of 10.8 mg every 3 months
- Arm 2: Apalutamide monotherapy
  - Apalutamide administered at a single 240 mg daily oral dose (4 × 60 mg tablets)
- Arm 3: Abiraterone acetate + Prednisone + Apalutamide
  - Abiraterone acetate administered at a single 1000 mg daily oral dose (4 × 250 mg tablets)
  - Prednisone administered at a 5 mg twice daily oral dose
  - Apalutamide administered at a single 240 mg daily oral dose (4 × 60 mg tablets)

Per protocol, study treatment is planned until week-25. Patients will be treated until objective or clinical disease progression or the occurrence of unacceptable toxicity. Patients are allowed to continue study treatment beyond week 25 (extension phase) at the discretion of the investigator.

Patients will be discontinued from the planned study treatment due to progression (radiographic per RECIST 1.1 and/or symptomatic +/− biochemical according to PCWG3 criteria), adverse event or patient withdrawal.

### Study outcomes and procedures

The primary endpoint of this study is to evaluate the proportion of patients who achieve an undetectable PSA level, defined as ≤0.2 ng/mL, at week 25 in each of three arms. Secondary endpoints are: determine and compare PSA progression rate at week 25 (PCWG3 criteria); determine and compare PSA response of 50 and 80% at week 25; determine maximum PSA declines and overall PSA change from baseline up to week 25 and up to week 52; determine the radiographic progression-free survival (rPFS) at week 25 among the three arms; determine and compare hormonal levels during treatment; determine and compare the safety profile; determine and compare the time to pain progression assessed by BPI-SF and opioid use; determine and compare the quality of life assessed by FACT-P; determine time to prostate cancer castration resistance; and metastasis-free survival (on non-metastatic patients at inclusion).

Table 1 shows the procedures which subjects will perform during all study phases. During the screening period, after signing informed consent, patients will undergo comprehensive clinical assessment, associated with cardiac evaluation (12 lead ECG and echocardiogram or MUGA). Following randomization, visits will be scheduled before each 28-day cycle, with evaluation of patient-reported outcomes, hematological and metabolic panel and PSA and testosterone levels. CT scans will be performed after 25 weeks, with a PSA confirmation at week 28. Afterward, patients who are benefiting from the study treatment, regardless of the treatment arm they have been assigned to, at week 25 are allowed to continue receiving this medication in an extension phase. These patients will be followed at 2 different times: 12 and 24 months after study treatment initiation. Biochemical and radiological progression and survival status data will be collected from the medical chart.

**Table 1:** SPIRIT flow diagram of LACOG 0415 phase II trial

Randomized study phase												Extension study phase
Cycle number	Screening	C1	C2	C3	C4	C5	C6	C7	PSA Confirmation	Safety visit	Patient chart data collection	Patient chart data collection
Week		0	4	8	12	16	20	25	28		Year 1	Year 2
Enrolment												
Eligibility screen	X											
Informed consent	X											
Medical history/clinical evaluation/vital signs/physical examination/ECOG/concomitant medication assessment	X	X	X	X	X	X	X	X		X		
12 lead ECG/Echocardiogram or MUGA	X							X				
Interventions												
Arm 1: Abiraterone + prednisona + Goserelin		X	X	X	X	X	X					
Arm 2: Apalutamide monotherapy		X	X	X	X	X	X					
Arm 3: Abiraterona + Prednisone + Apalutamide		X	X	X	X	X	X					
Study drug accountability		X	X	X	X	X	X	X				
Assessments												
CT scans								X				
Bone scan											X	
Hematology	X			X		X		X		X		
Biochemistry	X		X	X	X	X	X	X		X		
PSA	X		X	X	X	X	X	X	X			
Testoterone levels	X		X	X	X	X	X	X	X			
Patient reported outcomes (PRO)		X	X	X	X	X	X	X				
Survival Status											X	X

### Translational research

This study intends to collect biological materials (FFPE blocks or slides and blood samples) from patients and create a biorepository for translational research projects. The biorepository will comply with the current regulations in Brazil. Biomarker studies on the FFPE blocks or slides of tumor samples may include immunohistochemistry analyses, global miRNA profiling, tumor gene-expression profiling, and somatic mutational analyses.

Data collected from this study will be compared to historical data obtained from prior studies in advanced/metastatic hormone-sensitive prostate cancer to identify miRNA and GEP that correlate with the response (or primary resistance) to AAP and apalutamide. The biomarker results from this study will then be used to inform future studies of anti-androgen therapies possibly leading to product differentiation by selection of responsive subjects. Furthermore, we plan to investigate whether miRNA profiles may better define high-risk prostate cancer in the early advanced/metastatic disease setting. This data may then be utilized in the selection of high-risk patients in future studies if these previously reported miRNA profiles are confirmed and found to be more sensitive than conventional clinical estimates of high-risk disease.

### Statistical analysis

All statistical analyses specified in this protocol will be conducted using SAS version 9.4 and a significance level of 5%. For the primary endpoint (PSA below 0.2 ng/mL at week 25), and using Fleming one-stage method, a sample size of 38 participants per arm would allow 80% power to reject a PSA undetectable rate (defined as ≤0.2 ng/mL) of 45% or less, with an expected PSA response rate for each of the three arms of about 65% [15, 16]. All the efficacy analyses will take place using the intention to treat (ITT) population. No interim analysis for futility is planned.

Demographics and baseline disease characteristics will be analyzed using descriptive statistics. There is no formal statistical analysis plan for health-related quality of life (HRQOL). The FACT-P data will be scored and handled as recommended in its respective User’s manual [17]. Scores for each patient will be measured at baseline and every four weeks until week 25 and will be presented a spider-plot, without comparison between treatment arms. Differences greater than 10-points will be considered clinically significant [18]. The time-to-event endpoint will be estimated by Kaplan-Meier method and compared by stratified log-rank test or Cox regression method. Dichotomic data will be analyzed using Ficher’s exact test or Chi-squared test. If necessary, other methods for categorical data may also be applied as appropriate.

## Discussion

Management of locally advanced or metastatic prostate cancer still remains a clinical challenge. Androgen deprivation therapy remains the current standard of care, with high response rates in treatment-naïve patients, although most patients progress to castration-resistant prostate cancer [19]. Aside from its clinical efficacy, ADT is associated with adverse events that affect patients quality of life, as libido loss, sexual dysfunction, hot flushes, osteoporosis, muscle weakness and weight gain [1, 2]. Consequently, there is an unmet medical need for active treatments against the disease that can spare patients of testosterone suppression.

Apalutamide is a second-generation antiandrogen with a potent affinity for the AR but, unlike first-generation drugs, has no significant risk of agonistic activity [11]. In a phase II trial including 51 patients with non-metastatic castration-resistant prostate cancer, 89% patients had a biochemical response with a median time to PSA progression of 24 months, showing a robust activity with a tolerable safety profile [20]. Another phase II trial including 21 patients with castration-resistant prostate cancer who had failed prior abiraterone treatment has shown a response rate of 24% [21]. These data support the use of apalutamide monotherapy as an interesting option for patients with advanced/metastatic castration-sensitive prostate cancer. Additionally, as seen in the phase III SPARTAN trial [14], in men with nonmetastatic CRPC, apalutamide and ADT prolonged metastasis-free survival from 16.2 to 40.5 months (hazard ratio for metastasis or death, 0.28; 95% confidence interval, 0.23 to 0.35; *P* < 0.001). The more frequent adverse events as compared with placebo were rash, hypothyroidism, and fracture. There was no difference in androgen suppression-related side effects.

Abiraterone acetate is a selective inhibitor of androgen biosynthesis. It is currently approved in many countries in combination with prednisone, including Brazil and USA, for patients with metastatic castration-resistant prostate cancer based on two large randomized phase 3 clinical trials demonstrating a survival benefit for patients regardless of prior exposure to chemotherapy in metastatic setting [6–8]. Additionally, the recently published LATITUDE [9] and STAMPEDE [10] trials shown that the combination of AAP with ADT in patients with high risk metastatic castration-sensitive disease improves overall survival, leading to a use of this combination as a control arm in the present study. Moreover, abiraterone acetate plus apalutamide combination was evaluated in a phase IB [22] trial in 57 patients with progressive metastatic CRPC and shown a 67% PSA decline rate (≥ 50%) for patients who were abiraterone and enzalutamide-naïve. Conversely, in patients who were previously treated with those drugs, the rate was only 15%. Currently, there are two phase 3 trials active evaluating the combination for patients with metastatic (NCT02257736) or high-risk biochemically relapsed (NCT03009981) disease. Therefore, we hypothesized that the combination of AAP with apalutamide can at least provides the same benefit of the combination of AAP with ADT also possibly decreasing castration-associated side effects.

On the other hand, although two randomized trials have shown a survival benefit of adding docetaxel to androgen suppression in hormone naïve prostate cancer [23, 24], especially in patients with high volume disease, toxicity in both trials was higher in the chemotherapy/hormonal arms despite the survival benefit. Therefore, due to age and/or comorbidities when patients are not suitable candidates for chemotherapy, there is an urgent need to study and validate regimens such as new hormonal agents that may add benefit to castration with an acceptable safety profile.

One important limitation to consider in our study is that we chose the proportion of patients with PSA levels less than 0.2 ng/mL as our main endpoint because it is deemed a valid marker of prostate cancer treatment response. However, hormonal manipulation may change PSA levels without any significant difference in survival. Therefore, a phase 3 trial will be necessary even if our study is considered positive.

We expect that this study can answer the question if apalutamide as monotherapy or in combination with AAP is an effective hormonal treatment for patients with castration-sensitive advanced or metastatic prostate cancer, with acceptable safety profile, and possibly spare patients of androgen deprivation therapy side effects, as sexual dysfunction, osteoporosis, hot flushes, weight gain and increased cardiovascular risk.

## Appendix

**Table 2:** Details of all Ethics Committees which approved LACOG 0415 Protocol

INSTITUTION	ETHICS COMMITTEE	CITY	DATE OF APPROVAL
Hospital São José	HOSPITAL BENEFICÊNCIA PORTUGUESA DE SÃO PAULO	São Paulo	9/9/2016
Clínica AMO	FUNDAÇÃO BAHIANA DE CARDIOLOGIA – FBC	Salvador	6/20/2017
CEPHO	FACULDADE DE MEDICINA DO ABC\FUNDAÇÃO DO ABC - FMABC	Santo André	5/17/2017
CPO-RS	PONTIFÍCIA UNIVERSIDADE CATÓLICA DO RIO GRANDE DO SUL - PUC/RS	Porto Alegre	5/25/2017
CRIO	UFC - UNIVERSIDADE FEDERAL DO CEARÁ	Fortaleza	2/14/2017
Hospital do Câncer de Barretos	HOSPITAL DO CÂNCER DE BARRETOS / FUNDAÇÃO PIO XII	Barretos	1/26/2017
Oncologia D’Or	HOSPITAL PRÓ-CARDÍACO - ESHO EMPRESA DE SERVIÇOS HOSPITALARES	Rio de Janeiro	4/6/2017
COI	HOSPITAL PRÓ-CARDÍACO - ESHO EMPRESA DE SERVIÇOS HOSPITALARES	Rio de Janeiro	3/5/2017
ICESP	USP - FACULDADE DE MEDICINA DA UNIVERSIDADE DE SÃO PAULO - FMUSP	São Paulo	4/5/2017
Einstein	HOSPITAL ISRAELITA ALBERT EINSTEIN-SP	São Paulo	6/26/2017
Liga NG	LIGA NORTE RIOGRANDENSE CONTRA O CÂNCER	Natal	9/4/2018
Hospital Caridade Ijuí	UNIVERSIDADE REGIONAL DO NOROESTE DO ESTADO DO RIO GRANDE DO SUL - UNIJUÍ	Ijuí	10/8/2018
Hospital Erasto Gaertner	HOSPITAL ERASTO GAERTNER - LIGA PARANAENSE DE COMBATE	Curitiba	9/12/2018
IBCC	INSTITUTO BRASILEIRO DE CONTROLE DO CÂNCER - IBCC/ ONCOLOGIA CLÍNICA	São Paulo	9/11/2018

## Abbreviations

ADT: Androgen deprivation therapy
AR: Androgen receptor
FFPE: Formalin-fixed paraffin-embedded
GEP: Gene-expression profile
ITT: Intention to treat
miRNA: micro-ribonucleic acid
